# Hydrogen Sulfide Protects Against Ammonia-Induced Neurotoxicity Through Activation of Nrf2/ARE Signaling in Astrocytic Model of Hepatic Encephalopathy

**DOI:** 10.3389/fncel.2020.573422

**Published:** 2020-10-22

**Authors:** Xiaozhi Jin, Dazhi Chen, Faling Wu, Lei Zhang, Yu Huang, Zhuo Lin, Xiaodong Wang, Rui Wang, Lanman Xu, Yongping Chen

**Affiliations:** ^1^Department of Infectious Diseases, Wenzhou Key Laboratory of Hepatology, The First Affiliated Hospital of Wenzhou Medical University, Hepatology Institute of Wenzhou Medical University, Zhejiang Provincial Key Laboratory for Accurate Diagnosis and Treatment of Chronic Liver Diseases, Wenzhou, China; ^2^Department of Gastroenterology, The First Hospital of Peking University, Beijing, China; ^3^Department of Infectious Diseases and Liver Diseases, Ningbo Medical Center Lihuili Hospital, Ningbo, China; ^4^Department of Infectious Diseases and Liver Diseases, The Affiliated Lihuili Hospital of Ningbo University, Ningbo, China

**Keywords:** hepatic encephalopathy, primary astrocytes, H_2_S, blood microbiota, Nrf2

## Abstract

**Objective**: Hepatic encephalopathy (HE) characterized by neuropsychiatric abnormalities is a major complication of cirrhosis with high mortality. However, the pathogenesis of HE has not been fully elucidated. This study aimed to determine endogenous hydrogen sulfide (H_2_S) in the blood of HE patients and investigate the role of H_2_S in an astrocytic model of HE.

**Methods**: Patients with and without HE were recruited to determine plasma H_2_S levels and blood microbial 16S rRNA gene. Rat astrocytes were employed as a model of HE by treatment of NH_4_Cl. Exogenous H_2_S was preadded. Cell viability was measured by Cell Counting Kit-8 (CCK-8) assay, and cell death was evaluated by lactate dehydrogenase (LDH) release. Apoptosis was determined by Hoechst 33342/Propidium Iodide (PI) Double Staining and Western blot analysis of apoptosis-related protein expression. Intracellular reactive oxygen species (ROS) levels were assessed by flow cytometer. Expressions of Nrf2 and its downstream regulated genes were examined by immunofluorescence staining and Western blot, respectively. Nrf2 gene knockdown was performed by antisense shRNA of Nrf2 gene.

**Results**: There was a significant decrease in H_2_S levels in cirrhotic patients with HE compared with without HE. Blood microbiota analyses revealed that certain strains associated with H_2_S production were negatively correlated with HE. *In vitro*, H_2_S markedly attenuated NH_4_Cl-induced cytotoxicity, oxidative stress, and apoptosis. This effect was mediated by Nrf2/ARE signaling, and knockdown of Nrf2 expression abolished the antagonistic effect of H_2_S on NH_4_Cl-induced neurotoxicity in astrocytes.

**Conclusion**: Levels of H_2_S and bacteria associated with H_2_S production are decreased in HE, and H_2_S functions as the neuroprotector against NH_4_Cl-induced HE by activating Nrf2/ARE signaling of astrocytes.

## Introduction

Hepatic encephalopathy (HE) is a common complication of cirrhosis, leading to low quality of life and high mortality (Vilstrup et al., 2014). Feature of HE is a neuropsychiatric syndrome covering a broader range of disturbances including alterations in intellectual function, conscience, and motor function and coordination (Bustamante et al., 1999). Excessive ammonia generated by enteric bacteria crossing the blood-brain barrier and causing astrocyte swelling is a key driver of HE (Williams, 2007; Felipo and Butterworth, 2002). However, its pathogenesis is incompletely understood, and effective clinical treatments are still in development.

Hydrogen sulfide (H_2_S), a well-known cytotoxic gas, has recently been regarded as an important endogenous gasotransmitter, which contributes to physiological and pathological responses of various organs through antioxidant defense, energy production, and cell cycle regulation (Kadota and Ishida, 1972; Ortenberg and Beckwith, 2003; Kimura and Kimura, 2004; Poole, 2005; Lloyd, 2006; Yin et al., 2009; Henderson et al., 2010). In tissue and blood, H_2_S is kept in a range of concentrations to maintain physiological processes (Kamoun, 2004). Endogenous H_2_S generation relied on cystathionine-γ-lyase (CSE), cystathionine-β-synthase (CBS), and 3-mercaptopyruvate sulfur-transferase (Kimura, 2011). Furthermore, a significant amount of H_2_S in the host is derived from commensal bacteria (Rowan et al., 2009; Medani et al., 2011), which even profoundly controls tissue H_2_S bioavailability and metabolism along with alterations in synthesis enzyme activity and substrate availability (Shen et al., 2013). Increasing evidences have demonstrated a role of H_2_S in many neurological diseases, such as Alzheimer’s disease (AD; Vandini et al., 2019), Parkinson’s disease (PD; Hu et al., 2010; Tiong et al., 2010; Kida et al., 2011; Lu et al., 2012; Xie et al., 2013), stroke (Yin et al., 2013; Gheibi et al., 2014; Liu et al., 2016), and hyperhomocysteinemia (Zhao et al., 2018). Moreover, H_2_S has been involved in the antagonism of neurotoxins, including glutamate (Kimura and Kimura, 2004), methylmercury (MeHg; Yoshida et al., 2011; Han et al., 2017), cocaine (Frankowska et al., 2015), carbon tetrachloride (CCl_4_; Ci et al., 2017), and acrylonitrile (AN; Yang et al., 2018). Of note is the antioxidative stress effect of H_2_S in diseases (Hu et al., 2010; Yin et al., 2013; Yang et al., 2018); however, the role of H_2_S in the prevention of HE remains unclear.

Oxidative stress (OxS) plays a major role in brain injury in a patient with cirrhosis (Heidari et al., 2018; Swaminathan et al., 2018). During ammonia toxicity, nicotinamide adenine dinucleotide phosphate (NADPH) oxidase isoforms (Poznyak et al., 2020) and the mitochondrial permeability transition pore (Bai et al., 2001; Rama Rao et al., 2003) are the main sources for the reactive oxygen species (ROS). Astrocyte swelling induced by intracellular glutamine accumulation triggers OxS through an *N*-methyl-D-aspartic acid (NMDA) receptor- and Ca^2+^-dependent mechanism, and in turn, the activation of NMDA receptor and OxS enhances astrocyte swelling. This self-amplifying cycle between astrocyte swelling and OxS results in neuronal dysfunction, ranging from trivial lack of awareness to coma (Häussinger et al., 1994, Häussinger, 2006; Zielińska et al., 2003; Reinehr et al., 2007). Nuclear factor erythroid 2-related factor 2 (Nrf2), termed the master regulator of antioxidant responses, is a transcription factor found to be frequently dysregulated in OxS (Arefin et al., 2020). During OxS, the static binding between Nrf2 and Kelch-like ECH-associated protein 1 (Keap1) in the cytoplasm is dissociated, which allows the translocation of Nrf2 into the nucleus, and Nrf2 interacts with the antioxidant response element (ARE) to initiate the transcription of target genes to alleviate OxS (Shen et al., 2015). Interestingly, Nrf2 has been suggested as a crucial target of H_2_S and a vital mediator for H_2_S to attenuate OxS (Keum, 2011; Liu et al., 2012; Li et al., 2013; Yang et al., 2015). Therefore, Nrf2 might play an important role in the protection of H_2_S on HE.

In the current study, we determine plasma H_2_S levels and the microbiota associated with H_2_S production in the blood of HE patients. Meanwhile, we try to investigate the neuroprotective effect of H_2_S against ammonia toxicity to astrocyte and the role of the Nrf2/ARE signaling pathway in this putative cytoprotective function.

## Materials and Methods

### Ethics Statement

The clinical study protocol was approved by the Human Research Ethics Committee, the First Affiliated Hospital, Wenzhou Medical University, China. All participants or guardians signed the consent form in accordance with the Declaration of Helsinki. The animal experiments were approved by the Animal Ethics Committee and carried out in accordance with the established Guiding Principles for Animal Research.

### Study Subjects

All cirrhotic patients involved in this study were diagnosed through biopsy and/or radiological evidence.

We included cirrhotic patients with exclusion of individuals in coma (including HE grade 4); individuals on current or past specific treatment for HE; individuals with malignancy, heart failure, hematological or autoimmune diseases, and HIV infection; and individuals with organ transplants. The healthy controls exhibited no disease symptoms. Individuals who had consumed alcohol within 3 months, individuals with systemic antibiotics within 6 weeks, and individuals with yogurt/probiotic consumption within 2 weeks were also excluded. All participants were recruited from the First Affiliated Hospital of Wenzhou Medical University, and cirrhotic patients were divided into two groups according to with/without HE (Ferenci et al., 2002). The diagnosis of HE depended on clinical symptoms of brain dysfunction based on a careful and comprehensive neuropsychiatric evaluation addressing consciousness, orientation, cognitive function, and sensory and motor function, together with the knowledge of the patient’s history.

### Clinical Data Collection

All clinical data were collected through face-to-face interviews with hepatologists. All measurements and questionnaires were voluntary. Weight and height of all subjects were measured by an attending physician, and then the body mass index (BMI) was calculated. Blood ammonia, biochemistry, and coagulation index were determined at the hospital biochemistry laboratory of the First Affiliated Hospital of Wenzhou Medical University. The Model for End-Stage Liver Disease (MELD) score was calculated according to a previous study (Malinchoc et al., 2000).

### Chemicals and Reagents

NH_4_Cl (purity ≥99.5%) was obtained from Sinopharm Chemical Reagent Company (Shanghai, China). NaHS and 2′,7′-dichlorofluorescin diacetate (DCFH-DA) were purchased from Sigma–Aldrich (St. Louis, MO, USA). Antibodies against GFAP, cleaved caspase-3, Bax, Nrf2, GCLC, heme oxygenase-1 (HO-1), lamin B1, beta actin, GAPDH, and goat anti-rabbit IgG H&L were purchased from Abcam (Cambridge, MA, USA). Antibodies against Bcl-2 were obtained from Affinity Biosciences (OH, USA). CCK-8 was purchased from Dojindo (Kumamoto, Japan). Lactate dehydrogenase (LDH) release assay kit was purchased from Jiancheng Bioengineering Institute of Nanjing (Jiangsu, China). Hoechst 33342/Propidium Iodide (PI) Double Stain Kit was obtained from Solarbio Science and Technology Company (Beijing, China). Human H_2_S Elisa kit was purchased from MSK Biotechnology Company Limited (Wuhan, China).

NaHS was used as an H_2_S donor. When NaHS is dissolved at pH 7.35–7.45, HS^−^ is released and binds to H^+^ to form H_2_S. This provides a solution of H_2_S at a concentration at about one-third of the original concentration of NaHS (Reiffenstein et al., 1992).

### Plasma Levels of H_2_S Assay

Plasma was prepared after blood collection, and H_2_S level was measured within 2 days after collection by employing a human H_2_S ELISA kit (Wuhan MSK Biotechnology Company Limited, China) with a microplate reader at a wavelength of 450 nm.

### Detection of Microbial 16S rRNA Gene in the Blood

Blood samples were collected and flash frozen. As reported previously (Païssé et al., 2016), microbial DNA was extracted. Universal primers linked with indices and sequencing adaptors were used to amplify the V4 regions of the 16S rDNA. Agencourt AMPure XP magnetic beads were used to purify the PCR amplification products and then labeled to complete the establishment of the library. The amplicon sequencing libraries were sequenced on an Illumina platform and clustered into operational taxonomic units (OTUs) for further microbial analysis.

### Identification of Bacteria Associated With H_2_S Production

Sulfur occurs in various oxidation states ranging from +6 in sulfate to −2 in sulfide. The pathway map of the Kyoto Encyclopedia of Genes and Genomes (KEGG)^1^ reveals that the oxidation states of sulfur from +6 to −2 (the production of H_2_S) are through the energy consuming assimilatory and the energy producing dissimilatory pathways. Gene information including the lineage was known by gene search on the National Center for Biotechnology Information (NCBI)^2^. Bacteria carrying the gene involved in the pathway above were identified as association with H_2_S production, and the identification was extended from species to higher taxonomic levels. For example, *Odoribacter splanchnicus* is known to produce H_2_S, and this designation was extended to all members of the *Odoribacter* genus (Nguyen et al., 2020).

### Primary Astrocyte Culture and Treatment

Postnatal 1- to 2-day Sprague-Dawley (SD) rats were provided by the Laboratory Animal Center of the First Affiliated Hospital of Wenzhou Medical University. Primary astrocytes were prepared from the cerebral cortices of SD rats as described previously (Yin et al., 2011; Yu et al., 2015; Yuntao et al., 2016). Briefly, cerebral cortices were freed of meninges by microscope, minced, dissociated with 0.125% trypsin (Gibco, Grand Island, USA) for 15 min, passed through sterile nylon sieves, and placed in Dulbecco’s modified Eagle’s medium (DMEM; Gibco, Grand Island, USA) with 10% fetal bovine serum (FBS; Gibco, Grand Island, USA) and 1% penicillin/streptomycin (Gibco, Grand Island, USA). After centrifugation at 500 *g* for 5 min, the cell pellets were resuspended and seeded on dishes. The culture was maintained at 37°C in a humidified 5% CO_2_/95% air incubator. The culture medium was changed every 4 days. Upon reaching confluence (10–12 days), cells were harvested for further research. Immunostaining revealed that >95% of cells were glial fibrillary acidic protein (GFAP)-positive astrocytes (Supplementary Figure 1).

In most experiments, confluent cells were detached from dishes and seeded into new dishes with complete culture medium, which were divided into four groups randomly: control, H_2_S, NH_4_Cl, and H_2_S + NH_4_Cl groups. The NH_4_Cl group was used as a cellular model of HE (Wang et al., 2018), where astrocytes were incubated in media with NH_4_Cl. Cells in the H_2_S + NH_4_Cl group and H_2_S group were pretreated with NaHS for 1 h, washed twice with phosphate-buffered saline (PBS), and then respectively incubated in complete culture medium with and without NH_4_Cl. This excludes the effect of H_2_S as direct reductant or oxidant scavenging action. Incubation time of cells in NH_4_Cl is depending on the approach before further analysis.

### Cell Viability Assay

Cell viability was measured with the CCK-8 assay. Astrocytes were seeded into a 96-well culture plate at a density of 1 × 10^4^ cells/well in 100 μl of culture medium overnight and then were treated as above for 24 h. CCK-8 solution (10 μl) was added to each well of the plate and incubated for 2 h in an incubator. Absorbance was measured at 450 nm using a microplate reader.

### Lactate Dehydrogenase Release Assay

LDH release assay (Decker and Lohmann-Matthes, 1988) was also performed to measure cytotoxicity. Briefly, after cells were treated as above for 24 h, cell medium was transferred into a 96-well plate, and LDH release kit was used to detect LDH release activity of damaged cells. Absorbance was measured at 450 nm.

### Assessment of Intracellular Reactive Oxygen Species Generation

Intracellular ROS levels were examined using the DCFH-DA staining method based on the conversion of non-fluorescent DCFH-DA to the highly fluorescent DCF upon intracellular oxidation by ROS. This cell-permeable fluorogenic probe is useful for the detection of H_2_O_2_, $O_{\text{2}}^{-}$, and OH^−^ and for the determination of the degree of overall OxS. At least 2 × 10^5^ cells in each group were treated as indicated for 4 h, and 10 μmol/l DCFH-DA in serum-free medium was added and incubated for 25 min at 37°C in the dark. DCF fluorescence was measured using a flow cytometer with excitation at 484 nm and emission at 530 nm.

### Detection of Apoptosis by Hoechst 33342/PI Double Staining

Hoechst 33342/PI Double Staining was used to detect cell apoptosis (Liu et al., 2016). Astrocytes were seeded at 2 × 10^4^ cells/well in 24-well plates. After the indicated treatments for 8 h, cells were stained with Hoechst 33342 and PI dye according to the manufacturer’s protocol. The percentage of apoptotic cells was observed using fluorescence microscopy.

### Western Blot Analysis

Total cellular protein was extracted from at least 5 × 10^6^ cells in each group using RIPA lysis buffer containing 1% phenylmethylsulfonyl fluoride (PMSF; Solarbio Science and Technology, Beijing, China), and the nuclear and cytoplasmic proteins were obtained with a nuclear and cytoplasmic protein extraction kit (Thermo Fisher Scientific, Waltham, MA, USA) according to the instructions of the manufacturer. Concentrations of protein from cells were determined by BCA protein assay reagent kit (Beyotime, Shanghai, China). After heat denaturation at 100°C for 10 min, proteins were loaded onto polyacrylamide gels (10–15%). Then, proteins were separated and were transferred to polyvinylidene fluoride (PVDF) membranes (Millipore, Billerica, MA, USA). The nonspecific proteins on membranes were blocked with 5% bovine serum albumin (BSA) for 2 h at room temperature. The membranes were incubated with appropriate primary antibodies against cleaved caspase-3 (1:500), Bcl-2 (1:2,000), Bax (1:1,000), Nrf2 (1:500), GCLC (1:2,000), HO-1 (1:1,000), beta-actin (1:10,000), lamin B1 (1:2,000), and GAPDH (1:10,000), respectively, at 4°C overnight, followed by incubation with the corresponding secondary antibodies (1:2,000) for 1 h at room temperature. The corresponding bands were detected using the Chemi Doc™ XRS þ imaging system.

### Immunofluorescence

Astrocytes were cultured in 24-well plates on glass slides at a density of 2 × 10^4^ cells/well with complete culture medium and cultured to 50–60% confluency. After treatment for 24 h, the cells were fixed in 4% paraformaldehyde for 15 min and then permeabilized with 0.3% Triton X-100 for 20 min. After blocking with goat serum for 2 h at 37°C, cells were incubated with primary antibodies against Nrf2 (1:500) at 4°C overnight. This was followed by incubation with goat anti-rabbit IgG H&L (1:1,000) for 1 h and counterstaining with DAPI (Thermo Fisher Scientific, Waltham, MA, USA). Cells were observed with a fluorescence microscope.

### Knockdown of Nrf2 Expression With Small Hairpin RNA

Adenovirus with antisense of Nrf2 small hairpin RNA (shRNA) was obtained from Genechem Company Limited, Shanghai, China). The sequences of the two shRNAs for Nrf2 target genes were as follows: shRNA1, 5′-aaGCAGCATACAGCAGGACAT-3′ and shRNA2, 5′-gaGCAAGAAGCCAGATACAAA-3′. Astrocytes were seeded in 6-well plates at a 50–60% confluence, and cells were transfected with adenovirus at a multiplicity of infection (MOI) of 100 for 5 h later, then supplemented with fresh medium, and continuously cultured for an additional 48 h. Transfection efficiency was confirmed by Western blot analysis of Nrf2 protein expression.

### Statistical Analysis

Data were expressed as median (interquartile range) or mean ± SEM. The comparisons of H_2_S levels, blood ammonia, ages, BMI, and MELD score were made using Kruskal–Wallis test. The correlation between plasma H_2_S level and HE grade was using Spearman. The comparison of the relative abundance of the HE and nonhepatic encephalopathy (NHE) patients was performed using the Wilcoxon rank-sum test. Multiple comparisons in astrocyte cultures were carried out using one-way ANOVA, Dunnett’s test, or the Student-Newman-Keuls (SNK) test as a *post hoc* test, as appropriate. All statistical analyses were performed using SPSS (22.0). Values of *P* < 0.05 were considered statistically significant.

## Results

### Characteristics of the Studied Groups

**Figure 1:**
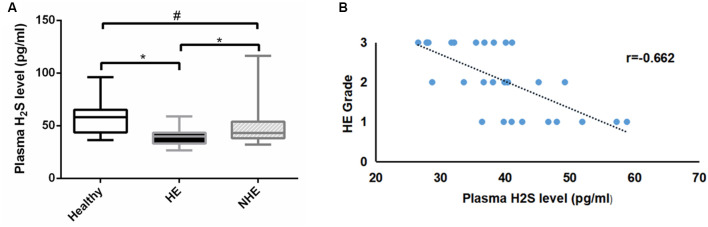
Plasma H_2_S levels. Panel **(A)** shows the plasma H_2_S levels in the healthy, hepatic encephalopathy (HE), and non-hepatic encephalopathy (NHE) groups. Panel **(B)** shows the correlation between plasma H_2_S level and HE grade. Data are presented as median (interquartile range). *P < 0.05, and ^#^*P* > 0.05.

A total of 89 subjects (healthy = 26, HE = 30, and NHE = 33) were studied. There was no difference in age or BMI among the three groups, nor was MELD score in HE and NHE patients (Table 1). As expected, the blood ammonia level in the HE group was significantly higher than that in the NHE group and healthy group (*P* < 0.001). Measurement of plasma H_2_S revealed a lower level in HE patients than in the NHE and healthy ones [39.8 (10.0) of HE vs. 43.2 (15.9) pg/ml of NHE or 54.1 (21.4) pg/ml of the healthy, *P* < 0.05; Figure 1A] and a negative correlation between plasma H_2_S level and HE grade (*r* = −0.662; Figure 1B).

**Table 1:** Characteristics of the study subjects.

Parameters	Healthy	Hepatic encephalopathy	Nonhepatic encephalopathy
	*n* = 26	(HE) *n* = 30	(NHE) *n* = 33
Ages (years)	54 (17)	58 (13)	62 (10)
Sex (M%)	92%	100%	94%
BMI (kg/m^2^)	21.9 (2.2)	23.5 (3.9)	23.2 (4.1)
Blood ammonia (μmol/L)	22.0 (19.3)	85.5 (36.5)	29.0 (28.5)
MELD score		20.4 (6.7)	16.5 (7.5)

*Continuous data are presented as median (interquartile range) and categorical data as n (%). MELD, Model for End-Stage Liver Disease*.

### Bacteria Associated With H_2_S Production in HE

**Figure 2:**
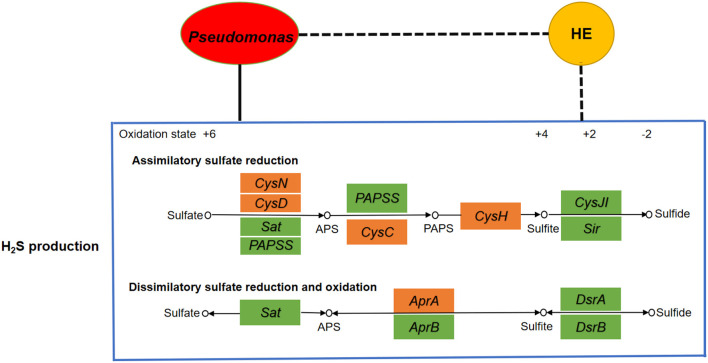
Analyses of correlation network among microbiota, H_2_S production, and HE. If the correlation is negative, the connecting line is black with dashes, whereas if positive, it is black. The yellow node represents hepatic encephalopathy, whereas the red one is the microbial community *Pseudomonas*. The big blue box shows the H_2_S production including assimilatory sulfate reduction pathway and dissimilatory sulfate reduction and oxidation pathway. The little boxes in green or orange represent genes, and the ones in orange are highly correlated with *Pseudomonas*. It clearly indicates a positive correlation between *Pseudomonas* and H_2_S production as well as a negative correlation between HE and H_2_S production.

Blood microbial 16S rRNA gene detection was performed in 22 HE and 29 NHE patients. Three taxa with significant different abundances between the HE and NHE groups were found to be association with H_2_S production. Genus *Staphylococcus* and phylum* Chloroflexi* were increased in the blood of HE patients; conversely, an enrichment of species *Pseudomonas alcaligenes* was detected in the NHE group. Among the genes implicated in the production of H_2_S, genus *Staphylococcus* carries genes *Sat* and *CysC*, and phylum* Chloroflexi* is only with gene *Sat*, whereas species *P. alcaligenes* negatively associated with HE carries the most genes related to H_2_S production, such as* CysH*, *CysD*, *CysN*, *CysC*, and *AprA* (Figure 2).

### H_2_S Attenuates NH_4_Cl-Induced Cytotoxicity in Primary Rat Astrocytes

**Figure 3:**
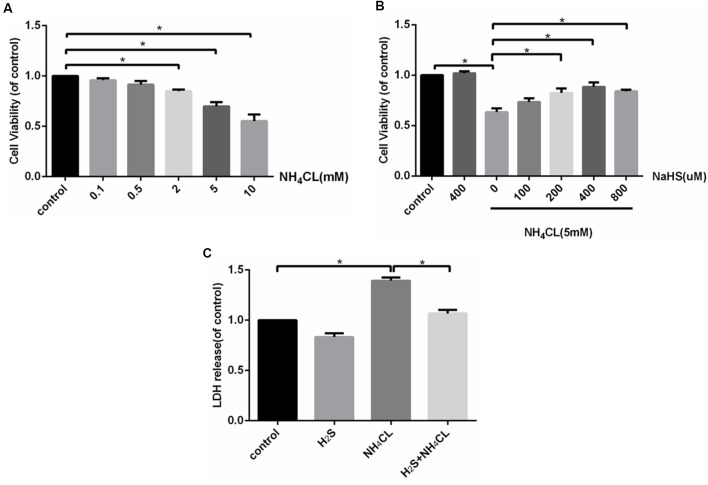
H_2_S attenuates NH_4_Cl-induced cytotoxicity in primary rat astrocytes. Panel **(A)** shows the effect of NH_4_Cl on astrocyte viability. Panel **(B)** displays the effects of NaHS pretreatment on NH_4_Cl-induced proliferation inhibition. Panel **(C)** indicates the cytotoxicity of astrocytes treated by control, H_2_S, NH_4_Cl, and combination of H_2_S and NH_4_Cl, respectively. Data are presented as mean ± SEM of three independent experiments. *P < 0.05.

The CCK-8 assay indicated that treatment with NH_4_Cl (2–10 mM) for 24 h significantly inhibited the proliferation of astrocytes in a concentration-dependent manner (*P* < 0.05; Figure 3A). Therefore, we used 5 mM NH_4_Cl to construct the HE models in follow-up studies. To assess the efficacy of H_2_S in attenuating the NH_4_Cl-induced cytotoxicity, astrocytes were treated with NH_4_Cl in the absence or presence of NaHS—an H_2_S donor. As shown in Figure 3B, the NH_4_Cl-induced decrease in cell viability was significantly attenuated by NaHS (200–800 μM; *P* < 0.05), and at 400 μM, NaHS exhibited the strongest effect. In addition, LDH in the cytoplasm is released if the cell is damaged. As shown in Figure 3C, pretreatment with NaHS (400 μM) significantly reduced NH_4_Cl-induced cell LDH release (*P* < 0.05).

### H_2_S Prevents NH_4_Cl-Induced Oxidative Stress in Primary Astrocytes

**Figure 4:**
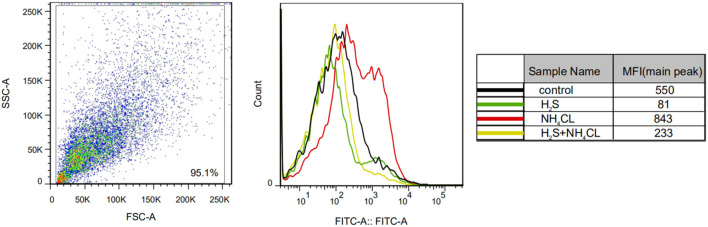
H_2_S prevents NH_4_Cl-induced reactive oxygen species (ROS) generation in primary astrocytes. ROS generation in astrocytes treated with control, H_2_S, NH_4_Cl, and combination of H_2_S and NH_4_Cl, respectively. The experiments were repeated three times. MFI, mean fluorescence intensity.

We examined the effect of H_2_S on NH_4_Cl-induced OxS in astrocytes by measuring ROS levels. As shown in Figure 4, NH_4_Cl significantly increased intracellular ROS generation compared with the control group (*P* < 0.05). Notably, pretreatment with NaHS significantly inhibited the NH_4_Cl-induced increase in ROS levels (*P* < 0.05).

### H_2_S Suppresses NH_4_Cl-Induced Apoptosis of Astrocytes

**Figure 5:**
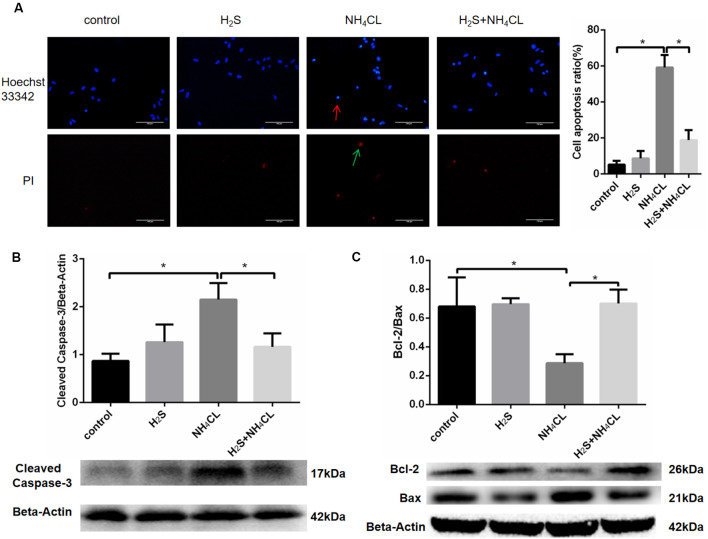
H_2_S suppresses NH_4_Cl-induced apoptosis of astrocytes. Panel **(A)** shows the immunostaining of astrocyte with Hoechst 33342 and Propidium Iodide (PI). Chromatin condensation in apoptotic cell is indicated by the red arrow, and necrotic cell stained in red is indicated by the green arrow, respectively. Cell apoptosis ratio (%) = The quantity of positive cells/The quantity of total cells. Panel **(B)** indicates the expression of cleaved caspase-3 in astrocytes treated with control, H_2_S, NH_4_Cl, and combination of H_2_S and NH_4_Cl, respectively. Panel **(C)** displays the expression of Bcl-2 and Bax in astrocytes treated with control, H_2_S, NH_4_Cl, and combination of H_2_S and NH_4_Cl, respectively. Data are presented as mean ± SEM of three independent experiments. *P < 0.05.

Double staining with Hoechst 33342 and PI was performed to investigate whether NaHS could mitigate astrocytic apoptosis induced by NH_4_Cl. Our result showed that NaHS significantly inhibited the apoptotic rate of astrocytes (*P* < 0.05; Figure 5A).

To further observe the antiapoptotic effect of H_2_S, the expression of apoptosis-related protein was detected by Western blot (Liu et al., 2016). Activation of caspase proteases has been considered as an important mechanism in apoptosis with caspase-3 accounted as an essential executioner. Western blotting demonstrated that the expression of active caspase-3 fragment (17 kDa cleaved caspase-3) was up-regulated by NH_4_Cl, and H_2_S significantly suppressed the NH_4_Cl-induced increase in cleaved caspase-3 level (*P* < 0.05; Figure 5B). Since pro- and anti-apoptotic members of the Bcl-2 family arbitrate the death or survival decision, the expressions of Bcl-2 (26 kDa) and Bax (21 kDa) were also examined. After treatment with NH_4_Cl, the expression of Bcl-2 decreased. In contract, the expression level of Bax increased. These observations were reversed by H_2_S (*P* < 0.05; Figure 5C). These results suggested that H_2_S suppressed NH_4_Cl-induced apoptosis of astrocytes through caspase-3 and Bcl-2 pathways.

### Activation of Nrf2/ARE Signaling Pathway Mediates the Protective Effects of H_2_S Against NH_4_Cl-Induced Neurotoxicity

**Figure 6:**
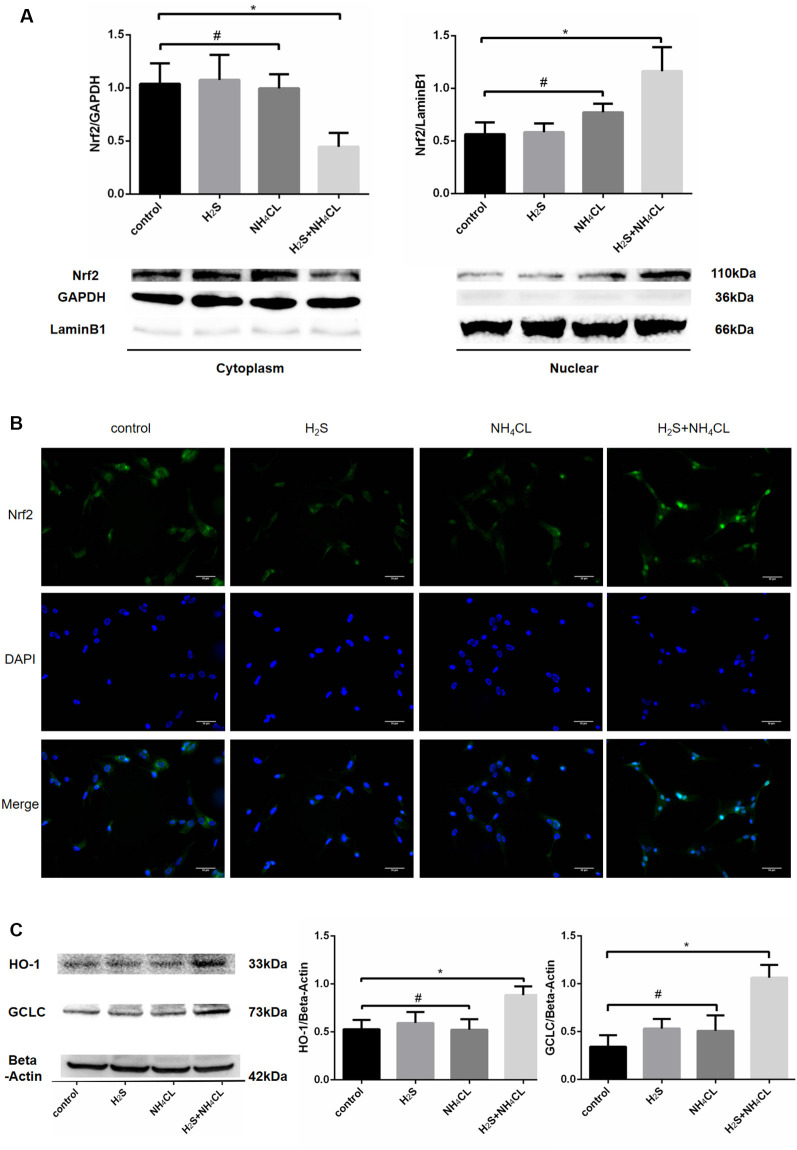
Activation of Nrf2 signaling by H_2_S during NH_4_Cl-induced toxicity in astrocytes. Panel **(A)** shows the expression of Nrf2 in the cytoplasm (left) and nucleus (right) with histograms on the top and typical Western blot pictures at the bottom. Panel **(B)** displays the immunostaining of Nrf2 and DAPI as well as merge pictures in astrocytes treated with control, H_2_S, NH_4_Cl, and combination of H_2_S and NH_4_Cl, respectively. Panel **(C)** indicates the expression of Nrf2 downstream genes HO-1 and GCLC treated with control, H_2_S, NH_4_Cl, and combination of H_2_S and NH_4_Cl, respectively, with typical Western blot picture on the left and histograms on the right. Data are presented as mean ± SEM of three independent experiments. *P < 0.05, and ^#^*P* > 0.05.

During OxS, Nrf2 enters the nucleus and binds to the ARE to initiate the antioxidant process. Western blotting analysis revealed a dramatical increase in the nuclear fraction of Nrf2 (110 kDa; *P* < 0.05) in the H_2_S + NH_4_Cl group, with a concomitant decrease in the cytoplasm (*P* < 0.05; Figure 6A). These results suggested that H_2_S could promote the translocation of Nrf2 from the cytosol to the nucleus. Similarly, immunofluorescence staining displayed that nuclear Nrf2 staining (green) was more abundant in the H_2_S + NH_4_Cl group than in any other groups where Nrf2 appeared to be mainly localized to the cytoplasm (Figure 6B). Moreover, the most essential downstream target genes of Nrf2 are HO-1 and GCLC. The expression of HO-1 (33 kDa) and GCLC (73 kDa) was significantly elevated in NaHS-pretreated cells after being exposed to NH_4_Cl (*P* < 0.05; Figure 6C). This provides further evidence that H_2_S activates the Nrf2/ARE signaling pathway against NH_4_Cl-induced neurotoxicity.

### Nrf2 Knockdown Blocks the Protective Effect of H_2_S in Primary Astrocytes

**Figure 7:**
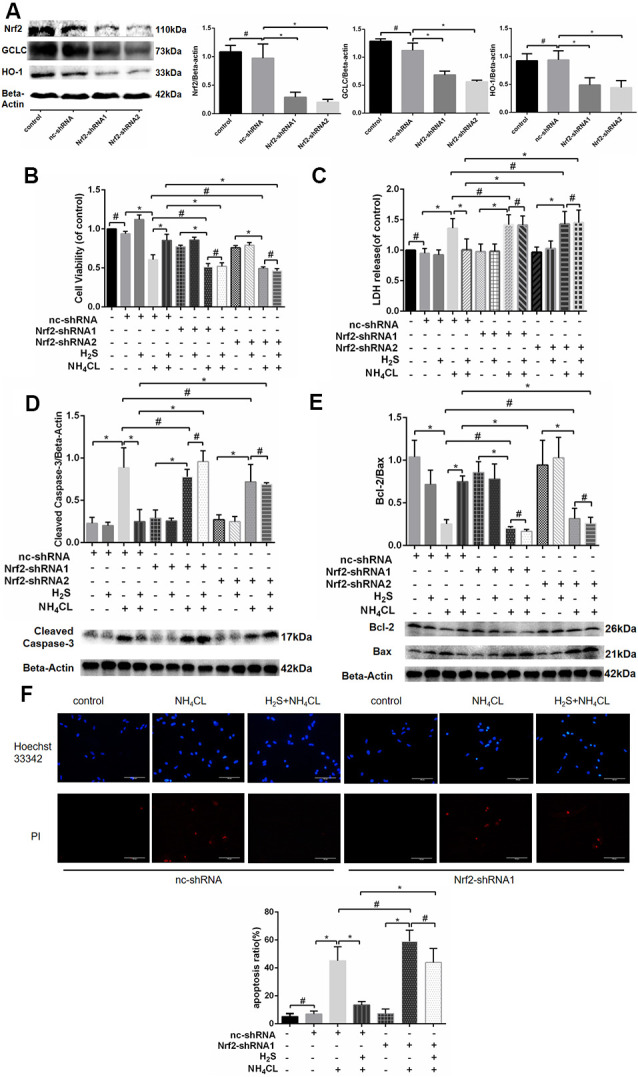
Effects of Nrf2 and H_2_S on astrocytes with and without NH_4_Cl treatment. Panel **(A)** shows that the antisense shRNA of Nrf2 attenuated the expression of Nrf2, HO-1, and GCLC in astrocytes with Western blot on the left and histogram on the right. Panel **(B)** shows the astrocyte viability, and panel **(C)** displays the lactate dehydrogenase (LDH) release in astrocytes treated with different combinations of control shRNA (nc-shRNA), antisense shRNA1 of Nrf2 (Nrf2-shRNA1), antisense shRNA2 of Nrf2 (Nrf2-shRNA2), H_2_S, and NH_4_Cl, respectively. Panel **(D)** indicates the cleaved caspase-3, and panel **(E)** shows the Bcl-2/Bax in astrocytes treated with different combinations of control shRNA (nc-shRNA), antisense shRNA1 of Nrf2 (Nrf2-shRNA1), antisense shRNA2 of Nrf2 (Nrf2-shRNA2), H_2_S, and NH_4_Cl, respectively, with Western blot at the bottom and histogram on the top. Panel **(F)** displays the immunostaining of Hoechst 33342 and PI in astrocytes under different cultures of control shRNA (nc-shRNA), antisense shRNA1 of Nrf2 (Nrf2-shRNA1), H_2_S, and NH_4_Cl, respectively, with staining picture on the top and histogram at the bottom. Data are presented as mean ± SEM of three independent experiments. *P < 0.05, and ^#^*P* > 0.05.

To further confirm whether Nrf2/ARE signaling was involved in the protective effect of H_2_S against ammonia toxicity to astrocytes, RNA inhibition was employed by shRNA1 and shRNA2 targeting Nrf2. As shown in Figure 7A, decreased expression of Nrf2 in astrocytes was observed at 27.54 ± 8.63 and 19.97 ± 6.15% relative to Nrf2 level in control after shRNA1 and shRNA2 treatments, respectively. Expressions of Nrf2 downstream genes HO-1 and GCLC were also down-regulated (*P* < 0.05; Figure 7A). Moreover, proliferation and apoptosis of astrocytes were examined. The proliferation of cells was inhibited, and they were more prone to apoptosis (albeit not statistically significant; Supplementary Figure 2). Furthermore, inhibition of Nrf2 significantly abolished the protective effect of H_2_S on the NH_4_Cl-induced decrease in cell proliferation (Figure 7B) and so to the NH_4_Cl-induced elevation of LDH release (Figure 7C). In addition, knockdown of Nrf2 expression blocked H_2_S protective effect on NH_4_Cl-induced apoptosis observed in both Hoechst 33342/PI staining and Western blotting (Figures 7D–F).

## Discussion

The results of the current study reveal the decrease of endogenous H_2_S and the effective protective role of H_2_S in HE astrocyte model. Our results suggest that: (i) there is a decrease in H_2_S production and bacteria associated with H_2_S production in the blood of HE patients; (ii) H_2_S attenuates NH_4_Cl-induced cytotoxicity, OxS, and apoptosis in primary rat astrocytes; and (iii) the Nrf2/ARE signaling pathway mediates the cytoprotection of H_2_S against NH_4_Cl-induced neurotoxicity.

Understanding of neurological disorder and H_2_S is emerging with more evidences suggesting that abnormal H_2_S generation can lead to neuronal dysfunction (Hu et al., 2010; Vandini et al., 2019). It is yet to be established whether HE characterized by neuropsychiatric abnormalities is associated with H_2_S synthesis. In the current study, the endogenous H_2_S levels were measured. Plasma H_2_S levels in HE patients were lower than those in cirrhotic patients without HE and healthy ones, and a negative correlation was found between the H_2_S level and HE grade. This suggests that the decline of H_2_S can promote neuronal dysfunction of HE disease. In addition, our results showed that H_2_S levels in the NHE group were lower than those in the healthy group, but statistically insignificant, indicating that the decrease of H_2_S is more closely associated with dysfunction of the brain than with the liver. Undoubtedly, a decrease in enzymes in the brain and liver is responsible for the reduction of H_2_S synthesis in HE. It is noteworthy that recent studies by Shen and colleagues found that the absence of microflora is connected with a significantly reduced CSE activity in many tissues coincident with an increase in tissue cysteine levels (Shen et al., 2013). These observations suggest an interesting hypothesis that bacteria could possibly influence enzyme activity or expression. A growing evidence indicated that a significant amount of H_2_S is produced by bacteria in the host. Systemic bioavailability and metabolism of H_2_S is also profoundly linked to bacteria (Rowan et al., 2009; Medani et al., 2011). Hence, HE in a patient with cirrhosis could be related to bacteria. In line of these findings, microbiota in the blood is mainly derived from gut microbiota and oral microbiota. Detection of blood microbial 16S rRNA gene, to our surprise, revealed that certain strains and functions of bacteria, especially *P. alcaligenes*, carry most genes related to the H_2_S generation but have negative correlation with HE. This suggested that certain strains associated with H_2_S production are reduced in HE, such as *P. alcaligenes*, and can up-regulate the H_2_S concentration by mediating the production of H_2_S. Thus, one of the main reasons for the decline of H_2_S in HE patients is the reduction of microbiota that can mediate the production of H_2_S.

H_2_S exhibits therapeutic efficacy in many neurological disorders (Hu et al., 2010; Gheibi et al., 2014; Zhao et al., 2018). Our results demonstrated that H_2_S synthesis and the microbiota associated with H_2_S production are decreased, however the role of H_2_S in HE remains unclear. Study on the role of H_2_S in astrocytic model of HE demonstrated that H_2_S mitigates NH_4_Cl-induced cytotoxicity, OxS, and cell apoptosis in primary astrocytes, which indicates its neuroprotective effect against HE.

Ammonia intoxication impairs mitochondrial function and activates NADPH oxidase, leading to the formation of ROS (Bai et al., 2001; Rama Rao et al., 2003; Poznyak et al., 2020). In the current study, the level of intracellular ROS increased significantly with ammonia treatment, suggesting that ammonia induces OxS in astrocytes. Previous studies have established that H_2_S works as an endogenous scavenger for ROS under OxS. Detection of intracellular ROS levels showed that H_2_S ameliorates the disrupted redox state induced by NH_4_Cl, characterized by decreased ROS.

Apoptosis is a physiological process of cell death and plays a critical role in many biological systems. It has been considered as an important molecular basis for ammonia-induced cell death (Wang et al., 2018; Zhang et al., 2018). Apoptosis induced by ammonia is regulated through a variety of signaling pathways, such as interruption of intracellular calcium ion (Ca^2+^) homeostasis and activation of the p53 pathway (Wang et al., 2018). Our results displayed that apoptosis of astrocytes was increased sharply with ammonia treatment, and that this was significantly attenuated by H_2_S. Recent studies on apoptosis popularly focused on OxS, and it has been reported that ROS can trigger apoptotic pathways (Chen et al., 2006; Wang et al., 2018; Zhang et al., 2018). In the present study, it displayed a consistent trend between apoptosis and ROS production, suggesting that apoptosis of astrocytes may be closely correlated with the overproduction of ROS. Based on these findings, we can conclude that H_2_S exerts neuroprotective effects by relieving astrocytic toxicity, OxS, and apoptosis against ammonia-induced HE.

H_2_S itself is a reductant that can neutralize free radicals. Recent studies have confirmed that the Nrf2/ARE signaling pathway plays a pivotal role in the antioxidative effect of H_2_S (Calvert et al., 2009; Yang et al., 2013; Liu et al., 2016; Kimura, 2014). During OxS, H_2_S can induce S-sulfhydration of Keap1, which contributes to Nrf2 dissociation from Keap1 and migration of Nrf2 into the nuclei to up-regulate the transcription of antioxidant genes (Calvert et al., 2009; Liu et al., 2016). Herein, results from both immunofluorescence and Western blotting analysis attested for the first time that pretreatment with NaHS contributes to nuclear translocation of Nrf2 and improves the expression of its downstream genes, such as GCLC and HO-1, against NH_4_Cl-induced neurotoxicity. When NH_4_Cl induces excessive ROS, H_2_S is able to prevent this oxidative damage by promoting nuclear translocation of Nrf2, which up-regulates the expression of a series of antioxidant enzymes, such as HO-1, to enhance detoxification and attenuate OxS. Our results showed that H_2_S does not increase nuclear Nrf2 expression by itself. This suggested that OxS is a prerequisite for activation of Nrf2/ARE signaling by H_2_S. In addition, knockdown of astrocytic Nrf2 quelled the protective effect of H_2_S in NH_4_Cl-induced cytotoxicity and cell apoptosis, which indicated that the Nrf2/ARE signaling pathway mediates the neuroprotection of H_2_S against HE.

Although the current study demonstrates a role of H_2_S in NH_4_Cl-induced HE astrocyte model and provides a new mechanistic explanation for the potential therapeutic value of H_2_S in the treatment of HE, there are still some limitations. The first limitation is the group division in patient study. Although the HE and NHE groups were divided based on clinical symptoms of brain dysfunction, patients with minimal HE have few recognizable clinical symptoms of brain dysfunction, which could lead to the possibility of patients with HE in the NHE group. The possibility could be minimized by excluding those currently or previously diagnosed with or suspected of HE in the NHE group. The second limitation is that patients in coma (including HE grade 4) were excluded since most coma patients experience hemodynamic instability or multi-organ dysfunction that could not reflect their “real” states, especially H_2_S levels. The third limitation is that the study could be better to have H_2_S level in cerebrospinal fluid (CSF); however, it is impossible to have CSF in patients with HE. Since there are evidences suggesting consistent H_2_S level between CSF and blood (Eto et al., 2002), the findings in the current investigation are still reliable. In addition, in the study of cell viability, NaHS showed the stronger effect at 400 μM than at 800 μM, indicating that high concentrations of NaHS might be cytotoxic and H_2_S exerts its neuroprotective effect within a proper range of concentrations.

In conclusion, the current investigation demonstrates that H_2_S acts as neuroprotection against NH_4_Cl-induced HE model by activating Nrf2/ARE signaling of astrocytes, and this contributes to the prevention of HE with the possible activation of the antioxidative defense system in astrocytes.

## Data Availability Statement

The sequence data have been deposited in the National Center for Biotechnology Information (NCBI) BioProject database with project number PRJNA640495 (https://www.ncbi.nlm.nih.gov/Traces/study/?acc=PRJNA640495).

## Ethics Statement

The studies involving human participants were reviewed and approved by the Human Research Ethics Committee, the First Affiliated Hospital, Wenzhou Medical University, China. The patients/participants provided their written informed consent to participate in this study. The animal study was reviewed and approved by the Animal Ethics Committee, Wenzhou Medical University, China.

## Author Contributions

LX and YC designed the protocol. XJ and DC performed the experiments and edited the manuscript. FW and LZ directed and participated in the sample detection. YH, ZL, RW, and XW analyzed the data. XJ wrote the manuscript, which was also edited by DC. All authors contributed to the article and approved the submitted version.

## Conflict of Interest

The authors declare that the research was conducted in the absence of any commercial or financial relationships that could be construed as a potential conflict of interest.

## Footnotes

^1^https://www.genome.jp/kegg/pathway.html

^2^https://www.ncbi.nlm.nih.gov/gene

## Supplementary Material

SUPPLEMENTARY FIGURE 1 GFAP immunostaining of primary astrocytes (shown in green).

SUPPLEMENTARY FIGURE 2 Effects of Nrf2 on astrocytes. Panels **(A,B)** show the antisense shRNA of Nrf2 regulated astrocyte viability and LDH release. Panel **(C)** displays the immunostaining of Hoechst 33342 and PI in astrocytes of control, control shRNA (nc-shRNA), and antisense shRNA1 of Nrf2 (Nrf2-shRNA1) groups, respectively, with staining picture on the top and histogram at the bottom. Data are presented as mean ± SEM of three independent experiments. Panel **(D)** shows that the antisense shRNA of Nrf2 regulated the expression of cleaved caspase-3 and Bcl-2/Bax, respectively, with Western blot at the bottom and histograms on the top, respectively. ^#^*P* > 0.05.
