# Supplementary figures and images for: Hydrogen Sulfide Protects Against Ammonia-Induced Neurotoxicity Through Activation of Nrf2/ARE Signaling in Astrocytic Model of Hepatic Encephalopathy
Source: Front Cell Neurosci. 2020 Oct 22;14:573422. doi: 10.3389/fncel.2020.573422 (PMC7642620; doi:10.3389/fncel.2020.573422)

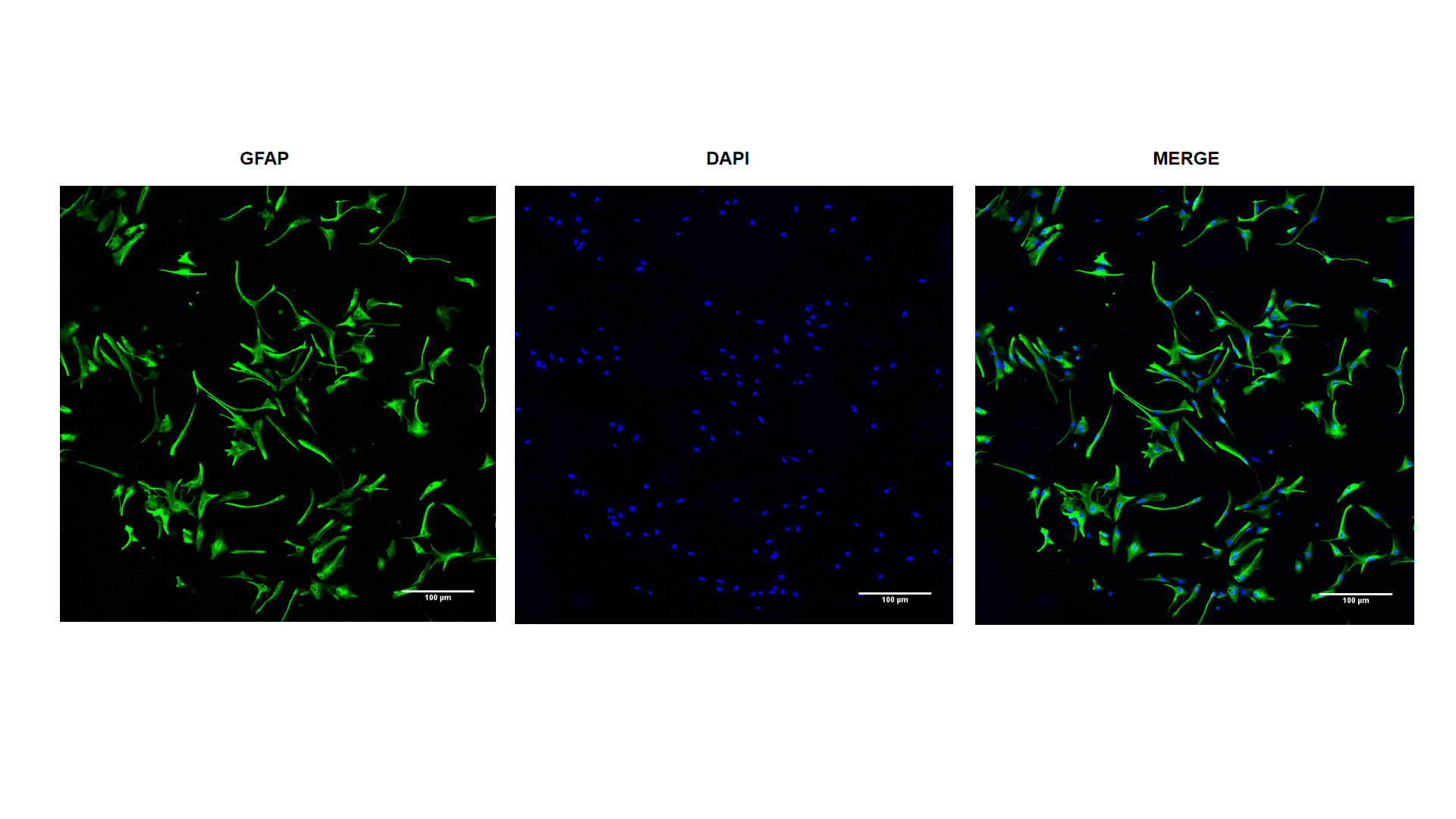

Supplement: SUPPLEMENTARY FIGURE 1 — GFAP immunostaining of primary astrocytes (shown in green). [file Image_1.TIF]

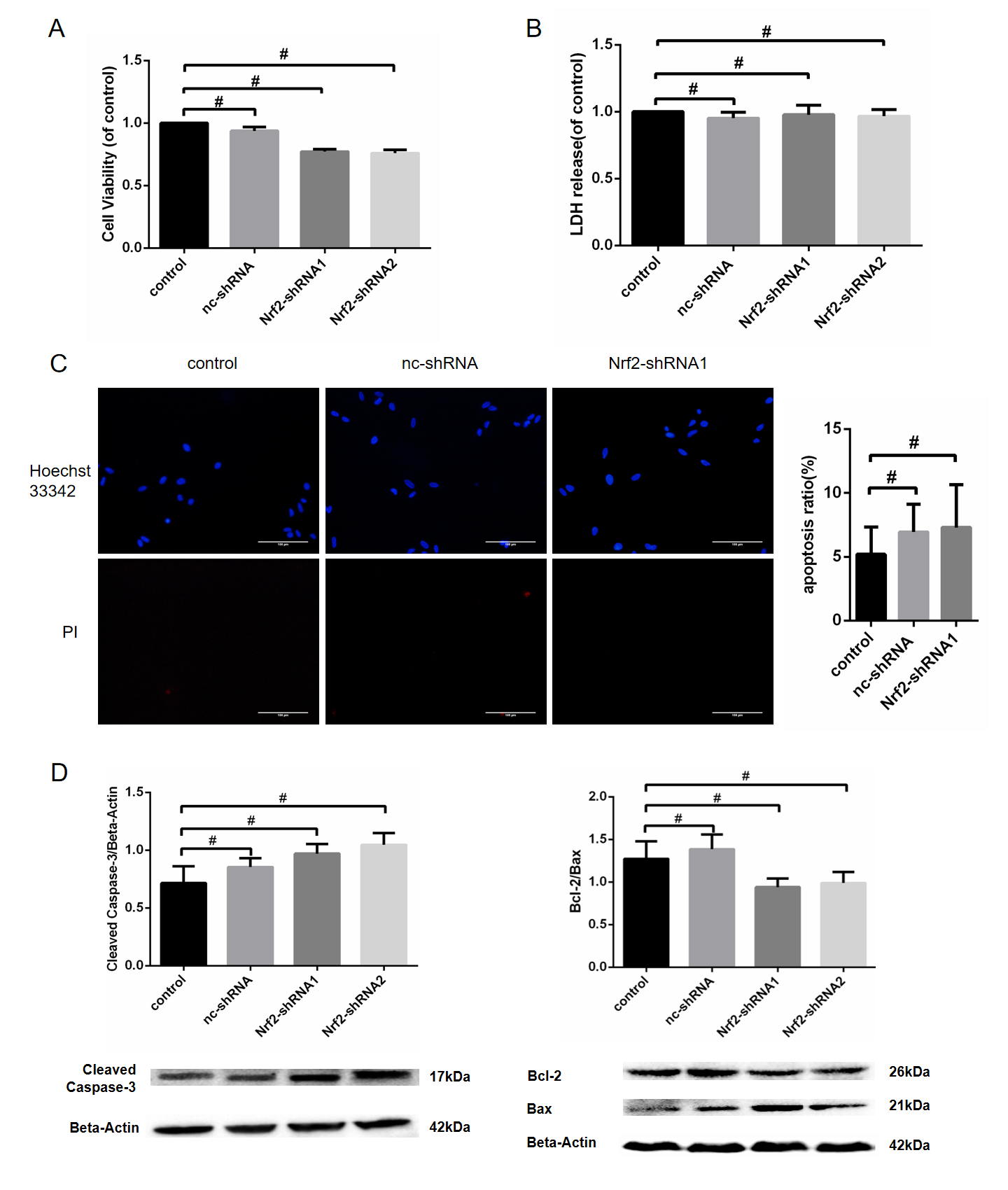

Supplement: SUPPLEMENTARY FIGURE 2 — Effects of Nrf2 on astrocytes. Panels (A,B) show the antisense shRNA of Nrf2 regulated astrocyte viability and LDH release. Panel (C) displays the immunostaining of Hoechst 33342 and PI in astrocytes of control, control shRNA (nc-shRNA), and antisense shRNA1 of Nrf2 (Nrf2-shRNA1) groups, respectively, with staining picture on the top and histogram at the bottom. Data are presented as mean ± SEM of three independent experiments. Panel (D) shows that the antisense shRNA of Nrf2 regulated the expression of cleaved caspase-3 and Bcl-2/Bax, respectively, with Western blot at the bottom and histograms on the top, respectively. #P > 0.05. [file Image_2.TIF]
